# Carbon dioxide induced plasticity of branchial acid-base pathways in an estuarine teleost

**DOI:** 10.1038/srep45680

**Published:** 2017-04-05

**Authors:** Elizabeth B. Allmon, Andrew J. Esbaugh

**Affiliations:** 1 University of Texas at Austin, University of Texas Marine Science Institute, Port Aransas, TX 78373, USA

## Abstract

Anthropogenic CO_2_ is expected to drive ocean pCO_2_ above 1,000 μatm by 2100 – inducing respiratory acidosis in fish that must be corrected through branchial ion transport. This study examined the time course and plasticity of branchial metabolic compensation in response to varying levels of CO_2_ in an estuarine fish, the red drum, which regularly encounters elevated CO_2_ and may therefore have intrinsic resilience. Under control conditions fish exhibited net base excretion; however, CO_2_ exposure resulted in a dose dependent increase in acid excretion during the initial 2 h. This returned to baseline levels during the second 2 h interval for exposures up to 5,000 μatm, but remained elevated for exposures above 15,000 μatm. Plasticity was assessed via gene expression in three CO_2_ treatments: environmentally realistic 1,000 and 6,000 μatm exposures, and a proof-of-principle 30,000 μatm exposure. Few differences were observed at 1,000 or 6,000 μatm; however, 30,000 μatm stimulated widespread up-regulation. Translocation of V-type ATPase after 1 h of exposure to 30,000 μatm was also assessed; however, no evidence of translocation was found. These results indicate that red drum can quickly compensate to environmentally relevant acid-base disturbances using baseline cellular machinery, yet are capable of plasticity in response to extreme acid-base challenges.

Anthropogenic CO_2_ emissions have been rising rapidly since the industrial revolution causing an increase in atmospheric CO_2_. This CO_2_ dissolves into oceanic surface waters, where it reacts with water to form bicarbonate (HCO_3_^−^) and protons (H^+^). Since the pre-industrial era, oceanic CO_2_ levels have risen by as much as 30% raising the CO_2_ partial pressure (pCO_2_) to 400 μatm, which has caused the pH of ocean water to drop by 0.1 units^1^,^2^,^3^,^4^. Estimates suggest that if current trends continue, oceanic pCO_2_ could reach 1,000 μatm by the end of the century, reducing surface water pH by 0.3–0.4 units^1^,^2^,^5^.

Ocean acidification has been shown to have numerous behavioral and ecological effects on marine fishes^6^,^7^,^8^,^9^,^10^,^11^,^12^,^13^. It is thought that these effects are the consequence of elevated blood HCO_3_^−^ that is the result of compensation to a respiratory acidosis^14^,^15^,^16^. It is generally accepted that marine fishes primarily compensate for a respiratory acidosis by transporting acid and base equivalents into the environment and plasma, respectively, through specialized gill ionocytes^16^,^17^,^18^. Apical transport of protons is thought to primarily occur through Na^+^ H^+^ exchangers NHE2 and NHE3^17^,^18^,^19^,^20^,^21^,^22^,^23^. This pathway is particularly effective for marine fishes owing to the steep inward Na^+^ gradient. Protons are produced from CO_2_ by cytoplasmic carbonic anhydrase (CA-c; 24,25,26), which also produces HCO_3_^−^. This HCO_3_^−^ is transported back into the plasma by the electrogenic Na^+^ HCO_3_^−^ co-transporter (NBC)^25^,^27^ (1 Na^+^: 3 HCO_3_^–^;^28^,^29^), which has the benefit of raising plasma HCO_3_^−^ thereby offsetting the increase in plasma CO_2_ and returning pH to baseline values. More recently, studies in elasmobranchs have highlighted the importance of V-type ATPase (VHA) translocation in compensating for alkalosis^30^,^31^. It is as yet unclear if similar translocation to the apical membrane may play a role in compensating for an acidosis in teleost fishes.

The resilience of marine fish species to the long-term environmental degradation caused by ocean acidification is dependent on a number of factors. While evolutionary adaptation to ocean acidification is a possible route for some fishes with short generation times, typical evolutionary processes are thought to be too slow to provide a tangible route to resilience for long lived species^32^. Instead, a major factor is thought to be the presence of resilient genotypes that may already exist within a species or population^32^,^33^. A second major factor is the phenotypic plasticity of a species, either within an individual or through transgenerational mechanisms, as this is hypothesized to extend the time for more standard evolutionary processes to occur^32^,^33^,^34^,^35^,^36^. Due to clear implications of ocean acidification for fish acid-base balance, understanding the baseline capacity and plasticity of acid-base pathways is particularly relevant.

Estuarine fishes potentially act as ecologically and environmentally relevant models for the study of the impacts of ocean acidification and other low level acid-base disturbances. Estuaries play important roles in the life cycles of many marine teleost species by providing shelter and food to larval and juvenile individuals. Additionally, the biogeochemical aspects of estuaries – including regular eutrophication that drives increased levels of microbial respiration – makes them susceptible to changes driven by ocean acidification^37^,^38^,^39^. Conversely, the regular diel and seasonal shifts of CO_2_ in estuaries may provide fishes that inhabit these areas with a degree of built in resilience to acid-base disturbances. Red drum (*Sciaenops ocellatus*) utilize estuaries extensively during their early lifecycle^40^,^41^,^42^ and can potentially act as model species to determine the physiological mechanism employed by marine teleosts to compensate for hypercapnia driven acid-base disturbances. As such, this study aimed to characterize the physiological response of red drum to varying levels of respiratory acidosis. The first objective was to characterize the time course of whole animal acid excretion in response to increasing levels of hypercapnia. A second objective was to assess the short-term and long-term physiological plasticity in response to both environmentally relevant and physiologically extreme hypercapnia scenarios.

## Results

### Series I: Time course of acid excretion following hypercapnia exposure

**Figure 1:**
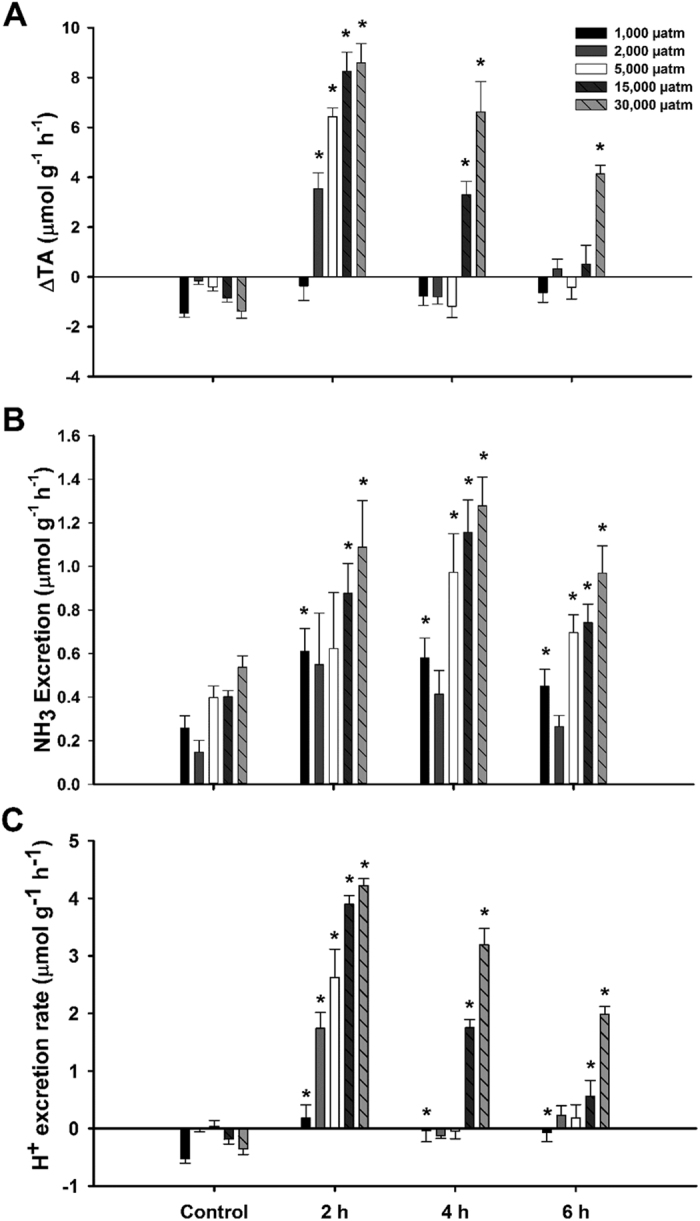
(A) Changes in titratable alkalinity, (B) Ammonia excretion, and (C) Net H^+^ excretion rates of red drum during control period and during 6 h post exposure to 1,000, 2,000, 5,000, 15,000 or 30,000 µatm CO_2_. Significant differences from controls denoted by an asterisk (ANOVA, P < 0.05). All values are mean ± S.E.M. 1,000 µatm n = 6, 2,000 µatm n = 5, 5,000 µatm n = 12, 15,000 µatm n = 6, 30,000 µatm n = 6.

During the 16 h period, all doses - except the 5,000 μatm dose - exhibited whole base excretion – as indicated by negative net H^+^ excretion (Fig. 1C). The 5,000 μatm dose exhibited slight net acid excretion but was not significantly different from zero (one sample t-test). All doses of CO_2_ significantly increased net H^+^ excretion by 2 h post exposure. Net H^+^ excretion in the 1,000 μatm dose was significantly increased at all time points compared to the control period; however, excretion was not significantly different than zero at any time point (one sample t-test). The 2,000 μatm and 5,000 μatm CO_2_ doses returned to control excretion rates by 4 h post exposure. In both the 15,000 μatm and 30,000 μatm CO_2_ doses, net H^+^ excretion remained elevated throughout the time series, although in both cases excretion rates appeared to be returning to control levels. While the vast majority of net H^+^ excretion was attributable to titratable acid flux (Fig. 1A), there was also widespread significant increases in NH_3_ excretion when exposed to elevated CO_2_ compared to control values (Fig. 1B). There was no significant effect of 1 mM amiloride (mean ± S.E.M. = 1.35 ± 0.11) on H^+^ excretion versus DMSO controls (mean ± S.E.M. = 1.38 ± 0.08; data not shown) during a 6 h flux period at 30,000 μatm CO_2_. Prior acclimation to 30,000 μatm CO_2_ had no effect on net H^+^ excretion rate when re-exposed to 30,000 μatm CO_2_. As above, all individuals exhibited a net base excretion during the 16 h control period, which was followed by significant net H^+^ excretion during initial 2 h exposure to 30,000 μatm CO_2_. There was no difference in net change of H^+^ excretion rate during initial 2 h exposure to 30,000 μatm CO_2_ between individuals pre-exposed and novel exposed individuals (Table 1).

### Series II: Branchial acclimation following hypercapnia exposure

**Figure 2:**
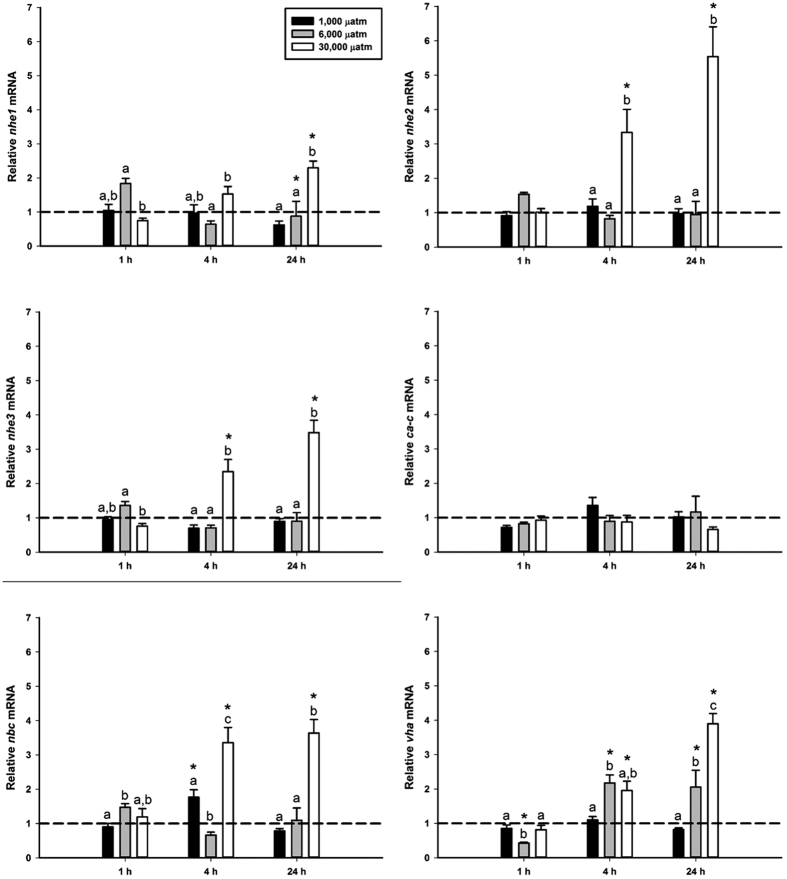
Gill expression of H^+^ excretion pathways during initial 24 h exposure to 1,000, 6,000, and 30,000 µatm nominal CO_2_. Values set relative to control values denoted by the dashed lines at 1.0. Significant differences from controls within CO_2_ dose denoted by an asterisk. Letters denote significant differences within each timepoint. All values mean ± S.E.M. n = 7–8.

**Figure 3:**
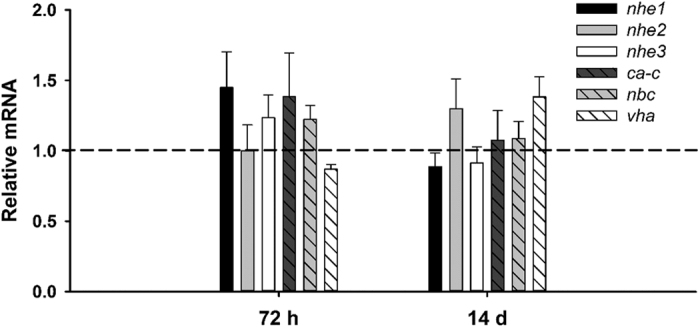
Gill expression of H^+^ excretion pathways during prolonged exposure (72 h and 14 d) to environmentally relevant 1,000 µatm nominal CO_2_. Values set relative to control values (dashed line at 1.0). All values mean ± S.E.M. n = 7.

Exposure to 1,000 μatm nominal CO_2_ induced changes in only one gene during the initial 24 h exposure. *nbc* was transiently upregulated at 4 h of exposure (Fig. 2). No changes were observed in expression of *nhe1, nhe2, nhe3, ca-c*, or *vha* during the 1,000 μatm CO_2_ exposure. Only *nhe1* and *vha* exhibited significant changes in expression during the 6,000 μatm CO_2_ exposure. At 24 h of 6,000 μatm CO_2_ exposure *nhe1* was significantly downregulated. At 1 h of 6,000 μatm CO_2_ exposure *vha* was significantly downregulated, followed by a significant upregulation at 4 h of exposure that remained upregulated throughout the course of the exposure. No changes in expression were observed during the 6,000 μatm CO_2_ exposure in *nhe2, nhe3, ca-c*, or *nbc* (Fig. 2). In contrast, the 30,000 μatm CO_2_ exposure induced significant upregulation of *nhe2, nhe3, nbc*, and *vha* as early as 4 h post-exposure, all of which remained elevated at 24 h exposure. *nhe1* expression was significantly elevated at 24 h post-exposure. No changes were observed in expression of *ca-c* at 30,000 μatm CO_2_ (Fig. 2). A final experiment assessed potential plasticity in response to more prolonged exposure to ocean acidification (1,000 μatm CO_2_); however, there was no significant effect at either 72 h or 14 d post-exposure (Fig. 3).

**Figure 4:**
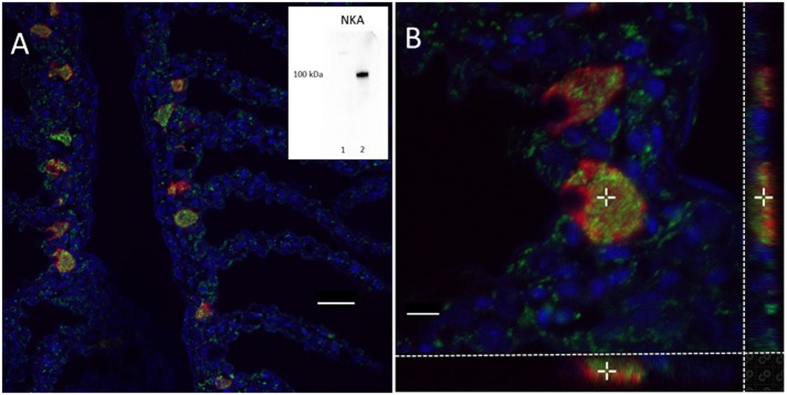
Immunofluorescent confocal micrographs showing branchial ionocytes. Red indicates Na^+^ K^+^ ATPase (NKA) and green indicates V-type H^+^ ATPase (VHA). Panel A: A representative image demonstrating the placement of ionocytes at the base of the secondary lamellae. Co-localization of NKA and VHA in ionocytes is demonstrated, but there is no evidence of sub-cellular co-localization (yellow). Additional VHA expression is found throughout the primary and secondary lamellae. Scale bar = 20 µm. Inset: Western blots demonstrating NKA antibody specificity in cytoplasmic (1) and membrane (2) protein fractions. Panel B: A 3-dimensional reconstruction of two ionocytes with showing x–y (main panel), x–z (lower panel, 50% scale) and y–z (side panel, 50% scale) orientations. The cross point is provided for orientation. No yellow color is demonstrated in any view point. Scale bar = 5 µm. One hour of 1,000, 6,000 or 30,000 µatm CO_2_ exposure had no effect on VHA localization (data not shown).

Immunofluorescence analysis of red drum gills verified that VHA was most abundantly expressed in ionocytes, as evidenced by co-localization with NKA, a known ionocyte marker (Fig. 4). Less abundant non-ionocyte expression was also apparent throughout the primary and secondary lamellae. While VHA and NKA were co-localized to the same cells, they did not exhibit sub-cellular co-localization under control conditions, as evidenced by the lack of overlapping fluorescence signal in 3 dimensional confocal reconstruction (Fig. 4B). These reconstructions suggest that VHA localization is mostly cytoplasmic, although some apical expression cannot be ruled out (Fig. 4B). No evidence of basolateral VHA localization was observed. To investigate the role of possible translocation to the apical membranes during compensation from an acidosis fish were exposed to 1,000 μatm, 6,000 μatm, and 30,000 μatm nominal CO_2_ for 1 h, after which VHA localization was again assessed. A 1 h time point was chosen to coincide with peak acid excretion, based on the results from *Series I*. There was no evidence of translocation of VHA to the apical membrane at any hypercapnia exposure (data not shown).

## Discussion

The results of this study largely support the conventional models with respect to marine acid-base balance in fish, and furthermore clearly demonstrate that red drum are quite effective at dealing with environmentally relevant acid-base disturbances (≤6,000 μatm CO_2_). While some degree of transcriptional change was observed in response to environmentally realistic disturbances, these responses were typically small and took place after acid excretion rates had returned to control levels. This would suggest that these responses play a relatively small role in compensation. These results are not entirely surprising as sub-adult red drum are estuarine-dependent, and therefore can routinely encounter diel shifts in environmental CO_2_ and pH levels. Nonetheless, more extreme disturbances resulted in wide-scale up-regulation of acid-base transporters demonstrating that acid-base regulation in red drum is responsive to environmental stress.

Our study of acid flux showed that the rate of H^+^ excretion by red drum increased sequentially with increased CO_2_ partial pressure. Red drum responded quickly to the imposed systemic acidosis as the first 2 h of exposure exhibited the highest rates of H^+^ excretion in all exposures. This supports previous work on a variety of marine species that have shown similar patterns^19^,^20^,^21^. After the 2 h time point, individuals exposed to the three lowest doses (1,000, 2,000, and 5,000 μatm CO_2_) had all returned to zero or control levels suggesting that they had fully compensated for the acid-base disturbance. This data supports previous work on blood chemistry in red drum whereby fish were fully compensated by 24 h after 1,000 and 5,000 μatm CO_2_^43^,^44^. A similar study on the gulf toadfish (*Opsanus beta*) further supports these findings by showing that the blood pH decreases significantly in response to hypercapnia (1,000 and 1,900 μatm CO_2_) as early as 15 minutes post exposure but returns to control levels within 2 h^14^. Interestingly, the excretion rates between the 15,000 and 30,000 μatm doses at 2 h of exposure are similar. This could be interpreted as a maximum excretion rate of the system, which is approximately 4 μmol g^−1^ h^−1^. Both the 15,000 and 30,000 μatm CO_2_ treatments remained elevated relative to the control period throughout the time trial; however, both exhibited a time-dependent trend of decreasing excretion rates towards control levels. Interestingly, the continued acid excretion suggests that the systemic acid-base disturbance was not fully compensated during the 2–4 h and 4–6 h intervals, yet the observed excretion rates were below maximum. This is likely related to a thermodynamic constraint on basolateral HCO_3_^−^ re-uptake from ionocytes caused by the elevated plasma HCO_3_^−^. Nonetheless, these trends suggest that even at high levels of hypercapnia, red drum are able to efficiently compensate for an acidosis. Despite the trends in gene expression at 30,000 μatm CO_2_ that suggest plasticity under extreme hypercapnic stress, repeated exposure to 30,000 μatm CO_2_ did not have an effect on red drum’s ability to excrete H^+^. Individuals that had been exposed to 30,000 μatm CO_2_ for 24 h exhibited the same apparent maximum excretion rate (4 μmol g^−1^ h^−1^) as those that had not been previously been exposed to elevated CO_2_. This suggests that the observed gene expression changes do not result in greater acid excretion capabilities; however, it is possible that they are required to maintain baseline function in the face of adverse intracellular conditions that may enhance protein turnover. Interestingly, NH_3_ excretion was significantly increased at the onset of elevated CO_2_ relative to control values. This is consistent with the ammonia trapping theory in which apical NHE function facilitates NH_3_ excretion^45^,^46^,^47^.

To more fully verify the predominant role of NHE proteins in net acid excretion, inhibition of acid excretion was attempted using the well-known NHE inhibitor, amiloride. However, inoculation with amiloride had no significant impact on net H^+^ excretion rates of red drum exposed to 30,000 μatm CO_2_. This is in contrast to experiments run on freshwater teleosts where amiloride successfully inhibited the H^+^ excretion pathways (primarily NHEs) in the gills^48^. While unlikely, it could be interpreted that these results suggest NHE is not a predominant route for apical H^+^ transport. A much more plausible explanation is that seawater may reduce the effectiveness of amiloride, either through reduced solubility or competition with Na^+^ 49. While increasing the concentration of amiloride within the experimental chambers may have had more success in inhibiting H^+^ excretion, amiloride becomes non-specific at higher concentrations – lessening the ability to accurately determine the source of excretion inhibition. These experiments highlight the difficulty in directly assessing the contributions of individual proteins to whole animal acid-base excretion.

The relatively few changes in gene expression found in the 1,000 μatm and 6,000 μatm CO_2_ exposures provide further evidence that red drum have adequate machinery in place to compensate for environmentally relevant acid-base disturbances. Of the genes tested, none showed consistent responses at either the 1,000 μatm or 6,000 μatm CO_2_ exposure. However, the transient upregulation of *nbc* during the 1,000 μatm CO_2_ exposure, may indicate that *nbc* is the limiting step in the compensation pathways. While it is possible that expression may have upregulated earlier than the earliest measured in this study, it is unlikely that the transcripts for these genes were increased and destroyed to return to control levels prior to the 1 h sampling point. A more plausible explanation is that red drum do not require upregulation of these genes until much later in the disturbance or until a higher level of disturbance has been reached.

An additional notable response was an observed down-regulation of *nhe1* at 24 h of exposure to 6,000 μatm CO_2_. It is well-known that *nhe1* is found on the basolateral membrane where it acts to move protect intracellular pH and contribute to plasma acidification by moving H^+^ from the cell into the plasma^17^,^50^. *nhe1* down-regulation is consistent with systemic acid-base balance in the face of an acidosis. It is somewhat surprising that neither *nhe2* nor *nhe3* showed any up-regulation when exposed to 1,000 or 6,000 μatm CO_2_, as these two proteins are thought to be crucial in acid-base compensation^17^,^18^,^21^,^23^,^51^. This is likely the result of sufficient baseline activity to account for these acid-base disturbances, which is supported by the whole animal acid flux measurements discussed above. Interestingly, a previous study showed that red drum *nhe2* and *nhe3* expression was also unchanged in the gill in response to freshwater transfer^52^. It is well-known that NHE proteins play a major role in Na^+^ uptake in freshwater and many euryhaline species show significant up-regulation in response to freshwater transfer^21^,^53^,^54^,^55^. The fact that red drum do not show up-regulation to this osmoregulatory stress – presumably a stress that would require more consistent NHE function than in seawater – is further evidence that these animals may simply maintain high NHE protein levels in their gills under normal circumstances. Nonetheless, the proof of principle 30,000 μatm CO_2_ treatment indicated that *nhe2* and *nhe3* – as well as *nhe1, nbc* and *vha* – are transcriptionally responsive to an acidosis.

Another noteworthy finding from the 6,000 μatm and 30,000 μatm CO_2_ treatments was the significant up-regulation of *vha*. While the involvement of VHA in freshwater acid-base compensation is well established^56^,^57^, this role is less certain for marine fishes. To date, most of the work examining VHA in marine acid-base compensation has focused on the translocation of cytoplasmic proteins to the basolateral membrane of ionocytes in response to an alkalosis^30^,^31^,^58^. However, it is important to note that this has not been demonstrated for a marine teleost. Our gene expression data suggest that VHA may play a role in H^+^ excretion across the apical membrane in marine fishes. VHA is strongly co-localized with NKA in the red drum gills, as there were no observable non-VHA ionocytes. This is somewhat different from previous results with longhorn sculpin that showed that VHA was only expressed in a sub-population of ionocytes^59^. The localization of VHA within the cell does not provide much functional insight as the protein is found largely in the cytoplasmic fraction, although it appears to be oriented toward the apical pole. Unlike previous work on alkalosis, there was no evidence of translocation of VHA to the apical membrane during hypercapnia at 1,000 μatm, 6,000 μatm, or 30,000 μatm CO_2_. While these results provide some support for the potential involvement of VHA in defending an acidosis, the lack of translocation or demonstrable apical localization suggest that VHA is not involved in apical H^+^ extrusion. Further work should examine whether VHA translocation may occur under more prolonged exposure scenarios as a complement to the primary NHE pathways.

In conclusion the combination of whole animal acid flux, gene expression and immunohistochemistry presented here provides a thorough description of the mechanisms and plasticity of acid excretion pathways in red drum following elevated CO_2_. Evidence of plasticity was only observed at extreme CO_2_ levels, while environmentally relevant concentrations and those associated with climate change do not result in plasticity and can be completely compensated within 2 h. This likely provides a physiological advantage in their estuarine habitat, where a number of factors can contribute to large shifts in ambient CO_2_ levels.

## Methods

### Animal Handling

Larval red drum were collected from Texas Parks and Wildlife hatchery in Corpus Christi, Texas. All fish were subsequently raised to the sub-adult stage at FAML. Fish were housed in recirculating systems with UV treated natural seawater collected from the Port Aransas ship channel with intermittent partial renewal. All tanks were aerated and ammonia was controlled by circulating water through a biofilter. Temperature was controlled using automated in-line heater/chiller units. CO_2_ was kept low through regular partial water replacement using ancillary flow-through lines. All fish were held on a 14 h:10 h light dark cycle. Fish were fed daily with commercially available Aquamax pelleted dry food and fish were acclimated to control conditions in the facility for two weeks prior to experimentation. All experiments were conducted in accordance with protocols approved by the University of Texas at Austin Institutional Animal Care and Use Committee.

### Whole Animal Acid Flux Measurements

Food was withheld from sub-adult red drum (n = 36, mean ± S.E.M = 15.0 ± 0.6 g) for 24 h prior to and throughout the experiment to avoid digestive/metabolic influences on acid-base regulation. Fish were placed in individual chambers (approximately 365 mL) with flow-through control seawater (salinity 32 ppt, temperature 22 °C, pH 8.12) and aeration. All individuals were given an 8 h chamber acclimation period prior to experimentation. After the acclimation period, the water source was removed and individuals underwent a 16 h control acid flux to establish normocapnia baselines. This was followed by a 1 h flush period prior to initiating hypercapnia. Preliminary experiments revealed that all significant acid flux occurred during the first 6 h (data not shown), and therefore hypercapnia exposures were limited to 6 h with acid flux measurements taken every 2 h. Hypercapnia exposures were initiated by removing the water source and switching aeration from ambient room air to room air mixed with nominal 1,000, 2,000, 5,000, 15,000, or 30,000 μatm (0.76, 1.52, 3.8, 11.4, 22.8 mmHg, respectively) CO_2_ (Table 2). A repeated exposure series at 30,000 μatm CO_2_ was also performed to assess whether increased gene expression conferred increased acid excretion rates. This series first exposed individuals to CO_2_ for 24 h followed by a 24 h recovery period under control conditions to return blood chemistry to control conditions. Immediately following the 24 h recovery period, acid flux experiments were performed as above with the exception that the flux period consisted of a single 2 h interval at 30,000 μatm CO_2_.

For all flux periods, water samples were taken at the beginning and end of the flux period for determination of titratable alkalinity and ammonia. The exact time of each sampling point was noted and pH was measured – calibrated with NBS buffers - at the onset of each flux period to verify CO_2_ treatment levels. Temperature, pH, titratable alkalinity and salinity measurements were also taken from the flush reservoir to validate CO_2_ partial pressures. At the conclusion of control and hypercapnia flux periods the individual chambers with water were weighed and the weight of the fish and empty chamber was subtracted to obtain the flux chamber volume. Immediately following water sample collection, each 25 mL flux period water sample was aerated with pure N_2_ gas for 15 minutes to remove any contribution of respiratory CO_2_ from the sample. Samples collected to verify CO_2_ treatment levels did not undergo N_2_ aeration. 10 mL of each sample was then titrated at 22 °C with 0.1 N HCl using an automated Apollo SciTech AS-ALK2 alkalinity titrator and accompanying software to determine titratable alkalinity. Ammonia concentrations were measured in each sample using a standard colorimetric assay^60^.

To elucidate the role of NHE’s in acid excretion, amiloride – a known NHE inhibitor – was introduced at the onset of CO_2_ exposure. Amiloride inhibition experiments were performed as above with the exception that experimental chambers were inoculated with 1 mM amiloride or 0.1% DMSO vehicle at the onset of hypercapnia exposure. Initial water samples were taken 15 min after drug inoculation and flux periods consisted of a single 6 h interval at 30,000 μatm CO_2_.

### Gene expression exposures

Sub adult red drum were held in 300 L recirculating tanks supplied with filtered, UV treated Port Aransas ship channel water (pH = 8.15) for at least two weeks prior to CO_2_ exposure. Fish were then transferred into a 300 L exposure tank or a 300 L control tank. Food was withheld for at least 24 h prior to sampling. Initial experiments were performed at control, 1,000, 6,000, and 30,000 μatm nominal CO_2_ for 4 h and 24 h (n = 8/treatment, mean body weight ± S.E.M = 39.5 ± 5.8 g). A second series performed 1 h exposures at all CO_2_ levels (n = 8/treatment, mean ± S.E.M = 29.6 ± 1.1 g). Each level of hypercapnia was maintained by aerating tanks through a gas-water equilibration tower with CO_2_ input controlled by gas mass flow controllers; control tanks were aerated with ambient room air. pCO_2_ exposure levels were calculated using CO_2_ Calc software^61^ using pH and titratable alkalinity measurements (Table 3). Immediately following exposure, specimens were euthanized with an overdose of tricane methanesulfonate (MS 222; 250 mg/l buffered with 500 mg/l NaHCO_3_) followed by spinal transection. Gill lamellae were excised and placed RNALater, and subsequently stored at −80 °C until processing. Gill arches were also collected from control and 1 h exposed fish and fixed in z-fix (Anatech Ltd.) overnight then stored in 70% ethanol until processing.

### Molecular Methods

Real time PCR primers were developed for *nbc* and *vha* (β-subunit), whereas primers for *nhe1, nhe2, nhe3, ca-c*, and elongation factor 1α (*ef1α*) had previously been developed for red drum^52^. Full length sequences for both *nbc* and *vha* were identified from an in-house gill/intestine transcriptome using Blaststation software. The identified sequences were then verified against the NCBI database using a standard Blast search. Primer pairs were identified using Primer3Plus software package^62^. All primers and GenBank accession numbers for related sequences can be found in Table 4.

Gill lamellae stored in RNALater were washed twice in 500 μL PBS before homogenization. Total RNA isolation was performed using TriReagent according to manufacturer protocols, and quantified using an ND-1000 spectrophotometer (Thermo Scientific). Total RNA was treated for potential DNA contamination by incubating with DNase 1 (Thermo Scientific), according to manufacturer protocols. cDNA synthesis was performed on 1 μg of total RNA using RevertAid M-Mulv reverse transcriptase (Thermo Scientific), according to manufacturer protocols. For all cDNA synthesis runs no reverse transcriptase controls were performed to test for genomic DNA contamination. Samples were diluted 10-fold using nuclease free water and stored at −20 °C until qPCR analysis.

qPCR analysis was performed using the Maxima SYBR Green kit (Thermo Scientific). Reactions were prepared according to the manufacturer’s protocols with the exception that a 12.5 μl total reaction volume was used. All reactions were processed using an MX3000 P qPCR machine (Stratagene) with accompanying software. A serial dilution was used for standard curves to determine the reaction efficiency of each primer pair. PCR efficiencies ranged from 74.2 to 100.8% with an R^2^ ≥ 0.97. For all genes, negative and no reverse-transcriptase control reactions were performed. The CT values for each sample were used to assess relative abundance of each gene in relation to the control gene *ef1α* using the delta-delta CT method^63^. Note that *ef1α* is a well-validated control gene for use in teleost fish^64^ that has been previously used in red drum^43^,^52^,^65^.

### Immunofluorescence Methods

Prior to staining, samples were dehydrated by three washes with 95% ethanol for 60 minutes followed by three washes with 100% ethanol for 45 minutes followed by a 1 h butanol wash and an overnight soak in butanol. Samples were then washed twice for 90 minutes in Histochoice clearing agent. Two paraplast washes were then conducted at 58 °C for 1 h before samples were set in paraffin and allowed to harden at room temperature. Samples were then stored at 4 °C until sectioning. Samples were sectioned at 20 μm and mounted onto Superfrost Plus slides where they were rehydrated, deparaffinised and soaked in DI water until ready to stain. Note that thick sections were used to allow for 3D confocal imaging of entire ionocytes. Each slide contained two sections so that no-primary antibody controls could be run concurrently with each sample. Antigen recovery was performed by heating slides in boiling 10 mM citrate buffer solution three times for 5 minutes. Hydrophobic barriers were drawn around each sample and samples were washed in blocking buffer (PBST with 5% fetal calf serum) twice for 5 minutes. Samples were then incubated with primary antibodies for NKA [1:100] and VHA [1:200] at 4 °C overnight. The primary polyclonal rabbit antibody for NKA (sc-28800) was obtained from the Santa Cruz Biotechnology and its effectiveness in red drum was verified by a Western blot that yielded only one band of approximately 100 kDa. The primary monoclonal mouse antibody for VHA was obtained from Santa Cruz Biotechnology and its effectiveness in red drum had previously been validated by our lab^65^. While there are commercial antibodies for NBC, NHE2 and NHE3, the target epitopes show low sequence similarity to red drum. Following primary incubation samples were washed in blocking buffer three times for 5 minutes then incubated with secondary antibodies – goat anti-rabbit Alexa Flour 555 [1:500] and goat anti-mouse Alexa Flour 488 [1:500] (Life Technologies) – in the dark for 1 h. Samples were then washed with blocking buffer three times for 5 minutes, stained with Sudan Black for 20 minutes to eliminate autofluorescence, and washed in PBS for 10 minutes. Finally, samples were washed with blocking buffer three times for 5 minutes and mounted using Vectashield with DAPI and stored in the dark at 4 °C until imaged. Imaging was completed using a Nikon C2+ confocal microscope system with a Nikon Eclipse Ti-E inverted microscope and utilizing NIS-Element imaging software for image acquisition, processing, and analysis.

### Statistical Methods

Gene expression data during the first 24 h of exposure to all treatments was assessed using a two-way ANOVA and Holm-Sidak post-hoc test using pairwise multiple comparisons. Prolonged exposures to 1,000 μatm CO_2_ had concurrently run control groups for 72 h and 14 d and thus were assessed by Student’s *t*-test against the respective control. All statistical tests were performed with a fiducial level of significance of p < 0.05. Acid-flux experiments were analyzed using one way repeated measures ANOVA with Holm-Sidak post-hoc test using multiple comparisons. Changes in H^+^ excretion during the amiloride exposure – against controls and between treatments – were analyzed using a two-way ANOVA with Holm-Sidak post-hoc multiple comparisons against controls.

### Ethical Approval

This article does not contain any studies with human participants performed by any of the authors. All applicable international, national, and/or institutional guidelines for the care and use of animals were followed. All procedures performed in studies involving animals were in accordance with the ethical standards of the University of Texas at Austin Institutional Animal Care and Use Committee (AUP-2015–00147).

## Additional Information

**How to cite this article**: Allmon, E. B. and Esbaugh, A. J. Carbon dioxide induced plasticity of branchial acid-base pathways in an estuarine teleost. *Sci. Rep.* **7**, 45680; doi: 10.1038/srep45680 (2017).

**Publisher's note:** Springer Nature remains neutral with regard to jurisdictional claims in published maps and institutional affiliations.

## Figures and Tables

**Table 1:** Net H^+^ excretion rates during control period and 2 h after initial and repeated exposures to 30,000 μatm CO_2_.

Treatment	Control	30,000 μatm	Δ net H^+^ flux
*Single Exposure to 30,000 μatm CO*_*2*_	−0.33 ± 0.09	3.97 ± 0.12	4.30 ± 0.18
*Repeat Exposure to 30,000 μatm CO*_*2*_	−0.72 ± 0.10	3.77 ± 0.24	4.49 ± 0.31

All rates mean ± S.E.M. μmol g^−1^ h^−1^.

**Table 2:** Water quality variables throughout acid flux experiments at varying levels of CO_2_.

Experiment	Treatment	Temp (°C)	Salinity (ppt)	pH	Alkalinity (μmol)	pCO_2_ (μatm)
*Acid flux*	1,000 μatm	22 ± 1	30 ± 1	7.81 ± 0.02	2195 ± 46	1074 ± 27
	2,000 μatm	23 ± 1	33 ± 1	7.59 ± 0.03	2134 ± 22	1756 ± 131
	5,000 μatm	23 ± 1	34 ± 1	7.15 ± 0.07	2160 ± 19	5373 ± 832
	15,000 μatm	23	32	6.69 ± 0.01	2318 ± 9	16281 ± 549
	30,000 μatm	24	32	6.33 ± 0.02	2327 ± 3	38317 ± 1466
*Pharmacology*	Amiloride	22	31	6.38 ± 0.07	2075 ± 10	31364 ± 4822
	DMSO	22	31	6.34 ± 0.06	2082 ± 13	33589 ± 4046
*Repeat exposure*	30,000 μatm	23 ± 1	32	6.37 ± 0.01	2211 ± 3	31459 ± 156

All values are mean ± S.E.M.

**Table 3:** Water quality variables for phenotypic plasticity studies.

Dose	Temp (°C)	Salinity (ppt)	pH	Alkalinity (μM)	pCO_2_ (μatm)
Control	23	32	8.15 ± 0.01	2,394 ± 17	477 ± 13
1,000 μatm	23	32	7.80 ± 0.01	2,348 ± 2	1,154 ± 35
6,000 μatm	23	32	7.13 ± 0.01	2,488 ± 13	6,181 ± 77
30,000 μatm	23	32	6.44 ± 0.01	2,346 ± 1	29,159 ± 364

All values are mean ± S.E.M.

**Table 4:** List of primers used for real-time PCR.

Gene	Accession #	Orientation	Sequence
ef1-a	**KJ958539**	F	GTTGCTGGATGTCCTGCACG
		R	GTCCGTGACATGAGGCAGACTG
nhe1	**KU899107**	F	AATGAGCTGCTGCACATCCTCG
		R	CAGACCACTCCGAGGACAGC
nhe2	**KJ958540**	F	CGGTTAAGCCTGATGGCCCTC
		R	TTGCAAACGAAGCCAGCAGC
nhe3	**KJ958541**	F	CAAGGTGCAGACCTTCACGCTG
		R	ACGAGGATGGCTCCCATGTT
ca-c	**KM387716.1**	F	TGACATTCGCAGACGACTCCGA
		R	AGCAGGATACTTGGTCCCTTCCA
nbc	**KM387714.1**	F	TCTTCATCTACGACGCTTTCAA
		R	TCATATTGAGTGACGAGGTTGG
vha	**KU899108**	F	CCTACCATTGAGCGTATCATCA
		R	CGTAGGAGCTCATGTCAGTCAG

All sequences are 5′ to 3′ and reverse primers are reverse compliments of the genetic sequence.
